# Characterization of clinical features and comorbidities between bipolar affective disorder with and without depressive episodes

**DOI:** 10.1017/S0033291722000782

**Published:** 2023-07

**Authors:** Chiao-Erh Chang, Jui Wang, Yi-Ting Lin, Chih-Chiang Chiu, Ming-Hsien Hsieh, Ming-Chyi Huang, Mong-Liang Lu, Hsi-Chung Chen, Wei J. Chen, Po-Hsiu Kuo

**Affiliations:** 1 Department of Public Health & Institute of Epidemiology and Preventive Medicine, College of Public Health, National Taiwan University, Taipei, Taiwan; 2 Department of Psychiatry, National Taiwan University Hospital, Taipei, Taiwan; 3 Department of Psychiatry, Taipei City Psychiatric Center, Taipei City Hospital, Taipei, Taiwan; 4 Department of Psychiatry, School of Medicine, College of Medicine, Taipei Medical University, Taipei, Taiwan; 5 Department of Psychiatry, Wan-Fang Hospital, Taipei Medical University, Taipei, Taiwan; 6 Center of Sleep Disorders, National Taiwan University Hospital, Taipei, Taiwan; 7 Center for Neuropsychiatric Research, National Health Research Institutes, Zhunan, Miaoli County, Taiwan

**Keywords:** Bipolar disorder, depression, physical conditions, psychiatric comorbidity, psychosocial factors, pure mania, unipolar mania

## Abstract

**Backgrounds:**

A proportion of patients with bipolar disorder (BD) manifests with only unipolar mania (UM). This study examined relevant clinical features and psychosocial characteristics in UM compared with depressive-manic (D-M) subgroups. Moreover, comorbidity patterns of physical conditions and psychiatric disorders were evaluated between the UM and D-M groups.

**Methods:**

This clinical retrospective study (*N* = 1015) analyzed cases with an average of 10 years of illness duration and a nationwide population-based cohort (*N* = 8343) followed up for 10 years in the Taiwanese population. UM was defined as patients who did not experience depressive episodes and were not prescribed adequate antidepressant treatment during the disease course of BD. Logistic regression models adjusted for relevant covariates were used to evaluate the characteristics and lifetime comorbidities in the two groups.

**Results:**

The proportion of UM ranged from 12.91% to 14.87% in the two datasets. Compared with the D-M group, the UM group had more psychotic symptoms, fewer suicidal behaviors, a higher proportion of morningness chronotype, better sleep quality, higher extraversion, lower neuroticism, and less harm avoidance personality traits. Substantially different lifetime comorbidity patterns were observed between the two groups.

**Conclusions:**

Patients with UM exhibited distinct clinical and psychosocial features compared with patients with the D-M subtype. In particular, a higher risk of comorbid cardiovascular diseases and anxiety disorders is apparent in patients with D-M. Further studies are warranted to investigate the underlying mechanisms for diverse presentations in subgroups of BDs.

## Introduction

Patients who experience manic symptoms are diagnosed with bipolar disorder (BD), which usually involves mood swings between depression and mania, the depressive-mania (D-M) type. However, there are a certain proportion of BD patients who only exhibit manic symptoms without depressive episodes, named unipolar mania (UM) (Baek, Eisner, & Nierenberg, 2014). The proportion of UM in the clinical BD patients varies widely across populations, from 1.1% to 65.3% (Mehta, 2014; Yazıcı, 2014). Besides potential differences in latitude, cultural factors, and genetic backgrounds, the lack of consensus among previous studies has been partly attributed to the absence of a standardized definition of UM.

The main requirement for UM is the absence of depressive episodes. However, the duration of follow-up and the minimum number of manic episodes required by different studies varies. The illness duration and follow-up periods were negatively correlated to the UM proportion. In most longitudinal studies, if the follow-up period was more than 5 years, the proportion of UM was lower than 15% (Angst, Gerber-Werder, Zuberbühler, & Gamma, 2004; Solomon et al., 2003; Stokes et al., 2020). Additionally, the reported proportion of UM has tended to be higher in non-Western countries, with averages of 40.8% *v.* 16.8% (Grobler, Roos, & Bekker, 2014). There have been few studies of UM among the Asian population. Earlier studies found a high proportion of UM (48.1%) in India and 36% among BD patients on lithium therapy in China (Lee, 1992; Rangappa, Munivenkatappa, Narayanaswamy, Jain, & Reddy, 2016). Moreover, the sources of BD patients from epidemiological or clinical samples also affect the proportion of UM as people tend to seek treatment for depression. A few epidemiological surveys, such as the National Comorbidity Survey (NCS) study, reported a high proportion of UM (39.7%) among adolescents and adults (32.67%) in the US (Angst et al., 2019; Merikangas et al., 2012).

Concerning clinical characteristics, patients with UM have been reported to have an earlier age of onset (Angst & Grobler, 2015; Yazıcı, 2014), more psychotic features (Grobler et al., 2014; Pfohl, Vasquez, & Nasrallan, 1982; Yazici et al., 2002), more cannabis abuse (Grobler et al., 2014; Pfohl et al., 1982), fewer suicide attempts (Angst et al., 2004, 2019), shorter sleep duration and an excessively hyperthymic temperament, a greater tendency to an early chronotype (morningness) (Angst & Grobler, 2015), and less comorbidity with anxiety disorders (Angst et al., 2019) compared with patients with D-M. These early observations were mainly obtained from clinical samples of European descent with relatively small sample sizes. Moreover, there has been an insufficient systematic evaluation of the differences between UM and D-M in terms of psychosocial factors, clinical factors, and psychiatric and physical comorbidities.

The co-occurrence of psychiatric and physical diseases is commonly observed in classic BD patients and has caused treatment and prognosis concerns in patient management. Particularly, certain psychiatric comorbidities (e.g. anxiety, substance use, and personality disorder) are correlated with worse cognition, more suicide attempts, frequent recurrence, and even higher rates of early mortality (Crump, Sundquist, Winkleby, & Sundquist, 2013; Kinrys et al., 2019; Vieta et al., 2001). This higher mortality is likely attributable to comorbid physical diseases in BD patients such as cardiovascular diseases, metabolic syndrome, endocrine and respiratory diseases (Goldstein et al., 2015; Sinha et al., 2018). Consequently, reduced life expectancy was observed in BD patients (Laursen et al., 2013). It is not yet well established whether the distribution of physical diseases varies between UM and D-M populations (Angst et al., 2019; Baek et al., 2014). This study aimed to evaluate patterns of comorbidity in UM and D-M patients, including both psychiatric and physical illnesses. We also aimed to describe the characteristics of UM that distinguish it from D-M.

## Materials and methods

### Study participants and data sources

**Fig. 1.**
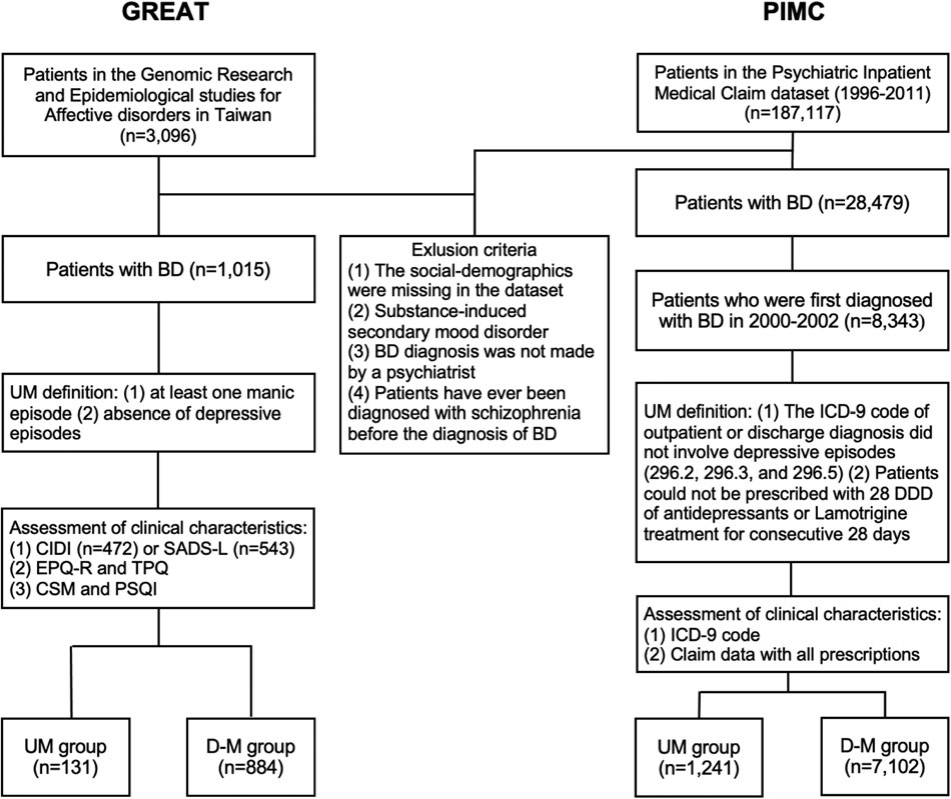
The patient selection flowcharts of GREAT study and PIMC cohort. BD, bipolar disorder; UM, unipolar mania; D-M, depressive-mania; DDD, define daily dose; CIDI, Composite International Diagnostic Interview; SADS, Schedule for Affective Disorders and Schizophrenia-Lifetime; EPQ-R, self-report Eysenck Personality Questionnaire-Revised; TPQ, Tridimensional Personality Questionnaire; CSM, Composite Scale of Morningness; PSQI, Pittsburgh Sleep Quality Index.

In the clinical retrospective study, patients were drawn from the database of Genomic Research and Epidemiological Studies for Affective Disorders in Taiwan (GREAT), which consisted of both family-based and case-control study designs. Subjects aged between 18 and 70 years were recruited between the year 2008 and 2020 from hospitals and the community. The current study used a subset of the GREAT data (i.e. BD patients only), in which patients diagnosed with BD according to the criteria of the Diagnostic and Statistical Manual of Mental Disorders, 4th Edition, Text Revision (DSM-IV-TR, American Psychiatric Association, 2000) were consecutively referred by psychiatrists in several collaborating hospitals in Taiwan. Exclusion criteria included patients who had ever received a diagnosis of mental retardation, schizophrenia, schizoaffective disorder, or substance-induced secondary BD. In total, there were 1015 patients with BD in the present study (Fig. 1). The median illness duration from onset to the time of recruitment was 9 years. The present study was approved by the institutional review boards of all participating hospitals, and all patients gave written informed consent.

Participants for the nationwide population-based cohort study were drawn from the Psychiatric Inpatients Medical Claim (PIMC) dataset, a part of Taiwan's National Insurance Research Database (NHIRD). The NHIRD includes data on more than 99% of the Taiwanese population and contains details of medical registration data, claim data, and demographic information. The PIMC involved all patients who had been hospitalized for any psychiatric diagnosis (*n* = 187 117). We identified BD patients by the International Classification of Diseases 9th revision Clinical Modification (ICD-9-CM, World Health Organization, 1996) codes 296.0, 296.4, 296.5, and 296.6 between 2000 and 2002 and followed up until the end of 2011 (Fig. 1). This gave us a longer period of follow-up to estimate the proportion of UM among those diagnosed with BD as illness duration is an important factor. The median follow-up years was 10.30 years. We included those who had received at least one inpatient diagnosis or two outpatient diagnoses of BD in order to ensure the validity of the diagnosis as previously suggested (Hsieh et al., 2019). Patients were excluded if they were diagnosed with a substance-induced secondary BD or had ever been diagnosed with schizophrenia before being diagnosed with BD. Our preliminary analysis (data not shown) for validation assessment of psychotic related diagnosis in the NHIRD revealed good validity in BD indicated by positive predictive values (0.796, 0.74–0.85) and sensitivity (0.841, 0.79–0.89) (Wang, 2021).

### Definition of UM

In the clinical GREAT cohort, UM was defined as those BD patients who self-reported at least one manic episode but no depressive episodes. The hypomania was not included as the current study focused on more extreme mood states (i.e. mania and depression). Patients who had experienced both manic and depressive episodes were classified as having D-M. D-M and UM patients were placed in two separate groups among all BD patients. Clinical information was collected using the modified Chinese version of the Composite International Diagnostic Interview (CIDI) or the modified Chinese version of the Schedule for Affective Disorders and Schizophrenia-Lifetime (SADS-L) (Huang et al., 2004; Kessler & Üstün, 2004).

For the PIMC cohort, we examined the medical records and discharge diagnoses to classify patients as UM or D-M. Patients with BD who met the following conditions were classified as UM: (1) The outpatient ICD-9-CM diagnostic code or discharge diagnosis did not involve depressive episodes (296.2, 296.3, and 296.5) or hypomania, which is not coded in ICD-9-CM. (2) The patient was not prescribed with a defined daily dose of antidepressants or lamotrigine for 28 consecutive days or more during the follow-up period.

### Measurement of clinical features, comorbidities, and psychosocial factors

Subjects of the GREAT study were interviewed face to face with the modified Chinese version of the CIDI (46.5% individuals) or the SADS-L (53.5%) to collect demographic characteristics, clinical data regarding mood episodes and symptoms, symptoms of psychosis, physical and psychiatric comorbidities, suicidal behaviors, etc. These questionnaires provided comorbidity information on autoimmune diseases (including arthritis and rheumatism), anxiety disorders, and substance use disorders (including smoking, alcohol use, and drug use). Both the CIDI and the SADS-L have good validity and reliability and have been widely used in epidemiological studies of different populations (Huang et al., 2004; Kessler & Üstün, 2004). The inter-rater reliability *κ* value was 0.77 for CIDI (Report of Taiwan Psychiatric Epidemiological Project) and 0.71–0.79 for SADS-L (Huang et al., 2004). The sensitivity of CIDI had been reported as 0.64 and specificity of 0.96 for depression with the Structured Clinical Interview for DSM-IV as the standard (Liao et al., 2012).

For the PIMC cohort, full records were available on clinical features and lifetime comorbidities. If patients had been diagnosed with ICD-9-CM code 296.X4, this indicated psychotic features to their BD. In cases of nonorganic psychosis, the ICD-9-CM codes used were 298, 297.8, and 297.9. Lifetime physical and psychiatric comorbidities were deemed admissible diagnoses if there were at least two outpatient records or one inpatient record with the diagnostic code for a given illness or condition. All diagnoses were classified using ICD-9-CM codes. Physical conditions found in the sample were type II diabetes (250), hyperlipidemia (272), thyroid disease (242, 244, 244.3, 244.8, and 244.9), cerebrovascular disease (430–438), hypertension (401–405), myocardial infarction (410), coronary artery disease (CAD, 411–414), renal disorder (582, 583, 585, 586, 588, and 590.0), chronic obstructive pulmonary disease (COPD, 491, 492, and 496), asthma (493), peptic ulcer disease (531–533), irritable bowel syndrome (564.1), constipation (564), autoimmune diseases (710 and 714), and epilepsy (345 and 780.3). The psychiatric comorbidities were personality disorder (301), all types of anxiety disorders (300.0, 300.2, and 300.3), generalized anxiety disorder (GAD: 300.02), panic disorder (300.01), phobic disorder (300.2), obsessive–compulsive disorder (OCD, 300.3), and substance use disorders (303–305).

In the GREAT study, we used the short Chinese version of the self-report Eysenck Personality Questionnaire-Revised (EPQ-R) and the short version of the Tridimensional Personality Questionnaire (TPQ) to measure personality traits. The Chinese version of the EPQ-R had good reliability of extraversion (0.79) and neuroticism (0.79). The Chinese version of the short-form TPQ has a high internal consistency of harm avoidance (0.87) and novelty seeking (0.70). We retained the four traits with good reliability in the present study. High *v.* low levels of the personality traits were defined using median scores in community control samples (Su et al., 2018). The cutoff point was set at 6.0 for trait extraversion, 4.0 for trait neuroticism, 7.0 for harm avoidance, and 7.0 for novelty seeking.

Chronotypes and sleep quality were assessed using the Chinese versions of the Composite Scale of Morningness (CSM) and the Pittsburgh Sleep Quality Index (PSQI) (Buysse, Reynolds, Monk, Berman, & Kupfer, 1989; Smith, Reilly, & Midkiff, 1989), respectively. Both of these have good reliability and validity (Gau, Soong, Lee, & Chiu, 1998; Tsai et al., 2005). The CSM was used to assess whether an individual is more alert in the morning or evening. Subjects were grouped into morningness (scores of 44 or higher), intermediate (scores of 23–43), and eveningness (scores of 22 or lower). The PSQI assesses sleep quality and sleep disturbances over the preceding month and comprises seven dimensions. The sum of these seven dimensions is the global score. The cutoff point for the Chinese version of the PSQI is a score higher than 5 for poor sleep quality.

### Statistical analysis

We used Student's *t* test and Pearson's χ^2^ test to examine differences between the demographics, clinical characteristics, and psychosocial factors of the UM and D-M groups. The Mann–Whitney *U* test was used where variables were not normally distributed. In addition, we performed multivariable regression analysis to evaluate the effects of relevant clinical variables on distinguishing the subtypes of BD, including illness duration, psychosis features, and suicidal ideation, while adjusted for sex and age. Logistic regression models were used to explore differences in effect size of lifetime comorbidities for physical diseases and psychiatric disorders between the UM and D-M groups. In the regression models, the odds ratio estimations (ORadj) were adjusted for sex and age in the GREAT study and for sex and age at the end of the study of the PIMC cohort. A two-sided *p*-value of <0.05 was considered statistically significant. All statistical analyses were conducted using SAS software version 9.4 (SAS Institute, Cary NC, USA). Data visualization was performed using R software version 4.0.2.

## Results

We first determined the proportion of UM among BD patients in the Taiwanese population using the clinical retrospective samples and nationwide cohort data (detailed information please see Fig. 1).

### Demographic and clinical characteristics of the UM and D-M groups

Table 1 displays the sociodemographic and clinical characteristics of the GREAT study participants. The average illness duration was approximately 11.64 ± 10.12 years and the average onset age was 28.25 ± 11.30 years. A total of 131 (12.91%) of the patients met the criteria for UM. More than 50% of patients had experienced at least three manic episodes. The UM group had significantly more symptoms of psychosis (44.71% *v.* 22.99%, *p* < 0.001), less suicidal ideation (39.69% *v.* 68.99%, *p* < 0.001) and fewer suicide attempts (11.45% *v.* 32.61%, *p* < 0.001) than did the D-M group. After adjusting for age, sex, illness duration, and clinical characteristics, we found that psychosis symptoms (ORadj 3.62; 95% CI 2.10–6.22), suicidal ideation (ORadj 0.27; 95% CI 0.15–0.47), and suicidal attempts (ORadj 0.32; 95% CI 0.14–0.77) remained significant to distinguish UM from D-M subgroups.

**Table 1.** Socio-demographic and clinical characteristics of UM and D-M groups in GREAT study

Variable	UM (*n* = 131, 12.91%)	D-M (*n* = 884, 87.09%)	*p*-Value
*Sociodemographic characteristics*			
Age of joining the study, mean (s.d.)^a^	41.45 (14.03)	39.74 (12.95)	0.112
Male gender, *n* (%)	66 (50.38)	390 (44.12)	0.179
Marital status, *n* (%)			0.814
Married	46 (35.11)	303 (34.28)	
Single	65 (49.62)	426 (48.19)	
Divorce or widowed	20 (15.27)	155 (17.53)	
Education year, *n* (%)			0.289
≤9 years (middle school)	20 (15.27)	146 (16.52)	
<16 years (high school)	73 (55.73)	429 (48.53)	
⩾16 years (college or higher)	38 (29.01)	309 (34.95)	
*Clinical characteristics*			
Illness duration (years), mean (s.d.)^a^^,^^b^	11.36 (10.45)	11.69 (10.08)	0.452
Age of first treatment, mean (s.d.)^a^^,^^b^	29.76 (12.43)	28.02 (11.10)	0.194
Age of first psychiatric hospitalization, mean (s.d.)^a^^,^^b^	30.93 (11.73)	31.70 (11.79)	0.524
First onset episodes, *n* (%)^b^			–
Manic episodes	131 (100)	529 (59.84)	
Depressive episodes	–	355 (40.16)	
Frequency of manic episodes, *n* (%)^b^			0.189
Once only	19 (20.43)	105 (27.13)	
2 times	22 (23.66)	61 (15.76)	
3 times	16 (17.20)	55 (14.21)	
⩾4 times	36 (38.71)	166 (42.89)	
Frequency of depressive episodes, *n* (%)^b^			–
Once only	–	117 (23.45)	
2 times	–	114 (22.85)	
3 times	–	83 (16.63)	
⩾4 times	–	185 (37.07)	
Psychosis symptoms, *n* (%)^b^^,^^c^	38 (44.71)	103 (22.99)	**<0.001**
Suicidal behaviors, *n* (%)			
Suicidal ideation	52 (39.69)	603 (68.99)	**<0.001**
Suicidal attempts	15 (11.45)	285 (32.61)	**<0.001**
Anxiety disorders, *n* (%)^b^^,^^d^	5 (11.63)	143 (34.36)	**<0.001**

*p*-Values in bold indicate significance at *p* < 0.05.

a The variable is not normally distributed, the test statistic uses the Mann–Whitney *U* test.

b Including missing value.

c Psychosis symptoms include hallucination, delusion, and disorganized thoughts. (The CIDI questionnaire lacked questions of psychosis symptoms).

d Anxiety disorders only include generalized anxiety disorder, panic disorder, and phobic disorder.

In the PIMC cohort, the average follow-up period was 9.83 ± 2.25 years and the proportion of UM was 14.87% (Table 2). The UM group was found to suffer less frequently from psychosis (*p* < 0.001), had fewer psychiatric hospitalizations (1.76 ± 2.09 *v.* 3.11 ± 4.36 *p* < 0.001), and less frequently utilized chronic psychiatric ward care (14.50% *v.* 17.63%, *p* = 0.007) when compared with the D-M group. A greater percentage of the UM group than that of the BD group was rediagnosed with schizophrenia during the follow-up period (27.88% *v.* 20.99%, *p* < 0.001).

**Table 2.** Socio-demographic and clinical characteristics of UM and D-M groups in PIMC cohort

Variable	UM (*n* = 1241, 14.87%)	D-M (*n* = 7102, 85.13%)	*p*-Value
*Sociodemographic characteristics*			
Age of joining the study, mean (s.d.)^a^	37.42 (15.65)	38.12 (15.21)	**0.043**
Follow-up periods (years), mean (s.d.)^a^	9.69 (2.51)	9.85 (2.20)	0.474
Male gender, *n* (%)	684 (55.12)	3279 (46.17)	**<0.001**
*Clinical characteristics*			
Anxiety disorders, *n* (%)^b^	257 (20.71)	3721 (52.39)	**<0.001**
Psychotic features, *n* (%)	481 (38.76)	3596 (50.63)	**<0.001**
Non-organic psychosis, *n* (%)	251 (20.23)	1323 (18.63)	0.185
Converting diagnosis to schizophrenia, *n* (%)	346 (27.88)	1491 (20.99)	**<0.001**
First episode type of joining the study, *n* (%)			–
Manic episode	1241 (100.00)	2220 (31.26)	
Depressive episode	–	4491 (63.24)	
Mixed episode	–	391 (5.51)	
Number of acute hospitalizations, mean (s.d.)^a^	1.76 (2.09)	3.11 (4.36)	**<0.001**
Episode type of hospitalization, *n* (%)^a^^,^^c^			–
Manic episode	2184 (100.00)	8305 (37.65)	
Depressive episode	–	12153 (55.09)	
Mixed episode	–	1602 (7.26)	
Between hospitalization intervals, mean (s.d.)^a^	771.48 (722.51)	584.79 (619.65)	**<0.001**
Ward type of hospitalization, *n* (%)			
Acute psychiatric ward	1020 (82.19)	6003 (84.53)	**0.038**
Chronic psychiatric ward	180 (14.50)	1252 (17.63)	**0.007**

*p*-Values in bold indicate significance at *p* < 0.05.

a The variable is not normally distributed, the test statistic used the Mann–Whitney *U* test.

b Anxiety disorders include any type of anxiety disorder.

c Exceeding the total number of samples, because it is the total number of hospitalizations for all patients.

### Physical comorbidities of the UM and D-M groups

**Fig. 2.**
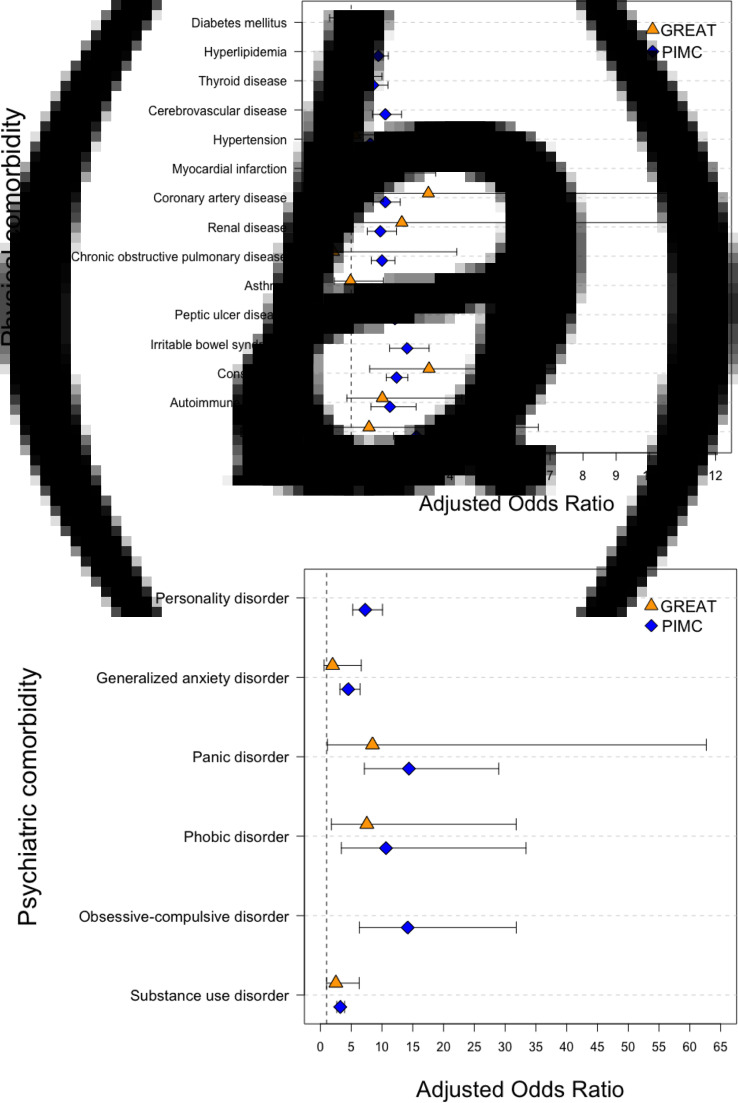
Estimation of effect size for comorbidity conditions between UM and D-M groups. (*a*) Physical comorbidity. (*b*) Psychiatric comorbidity.

Figure 2*a* shows the adjusted effect size estimations of lifetime comorbid physical conditions between the UM and D-M groups. In the GREAT study, the D-M patients were subject to significantly higher comorbid CAD (ORadj 3.33; 95% CI 1.03–10.78) and constipation (ORadj 3.35, 95% CI 1.56–7.17) in comparison with the UM patients, and these results were replicated in the PIMC cohort (ORadj 2.03; 95% CI 1.66–2.48 for CAD; and ORadj 2.37; 95% CI 2.06–2.71 for constipation). There was a significantly higher risk of all physical diseases but myocardial infarction among D-M patients in the PIMC cohort. In the GREAT study, the effect size estimations for comorbidity were not significant for any physical conditions besides CAD and constipation. This may have been due to the smaller sample size in this cohort.

### Psychiatric comorbidities of the UM and D-M groups

Figure 2*b* shows the adjusted effect size estimations of lifetime comorbid psychiatric disorders between the UM and D-M groups. In the GREAT study, D-M patients showed a significantly higher risk of comorbid panic disorder (ORadj 8.46; 95% CI 1.14–62.69), phobic disorder (ORadj 7.54; 95% CI 1.79–31.79), and substance use disorder (ORadj 2.51; 95% CI 1.00–6.29) when compared with the UM patients, and these results were replicated in the PIMC cohort. The D-M group showed a higher risk than did the UM group of generalized anxiety disorder in both the PIMC cohort (ORadj 4.52; 95% CI 3.17–6.44) and the GREAT study, but the difference did not reach statistical significance in the GREAT study (ORadj 1.98; 95% CI 0.59–6.63). The D-M group in the PIMC cohort had a higher prevalence when compared with the UM group of all types of anxiety disorders (ORadj 4.10; 95% CI 3.54–4.75).

### Psychosocial characteristics of the UM and D-M groups

We examined psychosocial factors in the UM and D-M groups in the GREAT study (Table 3). The UM group showed significantly higher extraversion, lower neuroticism, and less harm avoidance than the D-M group (*p* < 0.001). Significantly more of the UM group had a morningness chronotype than the D-M group (*p* = 0.017). The D-M group experienced significantly poorer sleep quality than the UM group (*p* < 0.001). The D-M group experienced more sleep disturbances (*p* = 0.022), more daytime dysfunction (*p* < 0.001), poorer subjective sleep quality (*p* = 0.003), and greater use of sleep medication (*p* = 0.022) than the UM group.

**Table 3.** Distribution of psychosocial factors between UM and D-M groups

Variable	UM (*n* = 131, 12.91%)	D-M (*n* = 884, 87.09%)	*p*-Value
Personality traits, mean (s.d.)			
Extraversion	6.54 (2.92)	5.37 (3.08)	**0.004**
Neuroticism	5.20 (2.28)	6.70 (2.22)	**<0.001**
Novelty seeking	7.80 (3.55)	8.27 (3.01)	0.240
Harm avoidance	7.94 (4.00)	10.66 (4.35)	**<0.001**
Chronotypes, *n* (%)^a^			**0.041**
Morningness type	22 (28.57)	62 (16.85)	**0.017**
Intermediate type	45 (58.44)	234 (63.59)	0.396
Eveningness type	10 (12.99)	72 (19.57)	0.176
Sleep parameters			
Total sleep time (h), mean (s.d.)	7.50 (1.96)	7.44 (3.66)	0.817
Sleep efficiency (%), mean (s.d.)	84.72 (18.96)	81.16 (24.04)	0.131
Sleep onset latency (min), mean (s.d.)	33.33 (30.28)	38.73 (34.46)	0.178
Greater than moderate level of sleep disturbance, *n* (%)	5 (5.88)	70 (15.18)	**0.022**
Greater than moderate level of daytime dysfunction, *n* (%)	11 (12.94)	143 (31.02)	**<0.001**
Use of sleep medication more than once per week, *n* (%)	53 (62.35)	343 (74.40)	**0.022**
Poor sleep quality, *n* (%)	18 (21.18)	176 (38.18)	**0.003**
Global PSQI score, mean (s.d.)	6.94 (3.84)	8.65 (4.06)	**<0.001**

PSQI, Pittsburgh Sleep Quality Index.

*p*-Values in bold indicate significance at *p* < 0.05. Both two groups have missing values for sleep parameters and chronotypes.

a This item transformed to *t*-score.

## Discussion

This study provides a more comprehensive characterization of the differences between UM and D-M groups in terms of sociodemographic, clinical, psychosocial characteristics, and lifetime comorbid physical illnesses and psychiatric disorders in an Asian population with two cohort samples.

In the Taiwanese population, the proportion of UM among BD patients was 12.91–14.87%, with slightly more males in the UM group (*p* < 0.001). The GREAT study used questionnaire-based interviews to record participants' experiences of depressive episodes. Since this method of data collection is reliant on patients' recall, there was some risk that episodes of mild depression might be forgotten. The PIMC data on depression was based on medical records of depressive episodes and antidepressant treatment of those patients who had been admitted to a psychiatric ward at any time. Therefore, the dataset may include relatively severe patients and bias the estimate of the true condition. Of note was the proportion of UM among BD patients in the present study slightly higher than those reported in Western countries such as the United States (5–12%) and Switzerland (6.4–13.6%) (Angst et al., 2019; Baek et al., 2014), but lower than those reported in non-Western countries such as China (36%), India (48%), and Tunisia (56–65%) (Amamou et al., 2018; Lee, 1992; Rangappa et al., 2016; Shulman & Tohen, 1994), all using clinical samples. Moreover, it is worth noting that clinical samples and claims data tended to underestimate the proportion of UM comparing to that estimated from epidemiological samples (Angst et al., 2019; Merikangas et al., 2012).

The proportion of UM among D-M populations varies between countries, possibly reflecting sunlight and latitude differences, ethnic and cultural differences (Amamou et al., 2018). Different criteria for UM in different studies are also likely to contribute to estimation differences. For example, the minimum number of manic episodes required is diverse across studies (Baek et al., 2014; Solomon et al., 2003; Stokes et al., 2020), the inclusion criteria for UM allowing patients who had experienced mixed episodes or some depressive symptoms (Perugi, Passino, Toni, Maremmani, & Angst, 2007), and differences in minimum illness duration and follow-up periods in previous studies. In the present study, we further conducted a sensitivity analysis to explore UM proportion estimates in different follow-up spans, i.e. follow-up 0–3 years, 4–10 years, and longer than 10 years. We found that the proportion of UM was 20.80, 14.70, and 14.50% for the three periods, respectively, indicating a relatively stable UM proportion estimate with follow-up longer than 4 years in our data.

In terms of clinical characteristics, we found more psychosis associated with UM in the GREAT study (*p* < 0.001). Although previous studies have reported more psychotic symptoms in UM groups than in D-M, they have not distinguished psychosis into psychotic symptoms or specific psychosis diagnoses (Mehta, 2014; Yazici et al., 2002). We further found that a higher proportion of UM than D-M patients was subsequently rediagnosed with schizophrenia (*p* < 0.001). Less suicidal ideation and fewer suicide attempts among patients with UM than with D-M were also noted in the present study. This was to be expected as suicidal behaviors are more likely to present in patients experiencing depression (Miller & Black, 2020). These results are in agreement with those found in other longitudinal studies and review articles that have demonstrated a distinct clinical profile for UM patients (Amamou et al., 2018; Angst et al., 2019; Grobler et al., 2014; Mehta, 2014; Yazıcı, 2014).

Previous research into physical comorbidities of UM and BD is scarce. We found substantial differences between the UM and D-M groups in lifetime comorbidities. The D-M group showed a higher risk of CAD and constipation when compared with the UM group in the GREAT study (*p* < 0.05). In the PIMC study, D-M patients showed a higher risk when compared with UM patients of all physical conditions (*p* < 0.05) but myocardial infarction. The GREAT clinical study had a smaller sample size and a younger average age than the PIMC cohort, which may account somewhat for the observed differences. In patients with classic BD, studies have found a two-fold increased risk of chronic physical conditions than that seen in healthy controls, including metabolic, cardiovascular, and gastrointestinal diseases, with ORs ranging from 1.4 to 2.4 (Crump et al., 2013; Scott et al., 2016; Sinha et al., 2018). On the other hand, BD is reported to have genetic overlap and shared biological mechanisms with metabolic and cardiovascular diseases (Amare, Schubert, Klingler-Hoffmann, Cohen-Woods, & Baune, 2017; Lu, Wang, Georgakis, Lin, & Zheng, 2021; Nowacki et al., 2020). Depression has also been widely reported to correlate with higher cardiovascular risks in population data (Carney & Freedland, 2017). The present study supported these findings. The PIMC cohort allowed us to examine a wide spectrum of comorbid physical illnesses, and this is the first study to report higher risks of renal, respiratory, gastrointestinal, autoimmune, and neurological diseases in D-M than UM populations. Several potential pathological pathways have been hypothesized to contribute to physical conditions in patients with BD, including oxidative stress (Wang, Chiang, Chen, & Shen, 2018) and common immunological abnormalities such as higher levels of proinflammatory cytokines in both bipolar and physical illnesses (Hsu et al., 2015; Rosenblat & McIntyre, 2015; Tsai et al., 2016).

For psychiatric disorders, we found that D-M patients have a higher prevalence than UM patients of comorbid generalized anxiety disorder, panic disorder, phobic disorder, and OCD in both the GREAT and the PIMC study. The odds of having any type of anxiety disorder were 4.10-fold higher in D-M than UM patients (Table 2). These results were in agreement with previous evidence (Andrade-Nascimento, Miranda-Scippa, Nery-Fernandes, Kapczinski, & Quarantini, 2011; Baek et al., 2014; Merikangas et al., 2012). It has been noted that comorbid anxiety disorders can be considered a severity marker in D-M and can influence disease course. It may also potentially identify which patients with mania will subsequently suffer from depressive episodes (Andrade-Nascimento et al., 2011; Otto et al., 2006). Research into the comorbidity of substance use disorder is less consistent. A meta-analysis from nine community samples indicated that UM patients are more likely to have comorbid drug use disorder than D-M patients (Angst et al., 2019). However, other studies have found that this is only true for amphetamine and cannabis abuse (Grobler et al., 2014; Pfohl et al., 1982). Contrary to previous research, we found a higher proportion of substance use disorder in the D-M group, both in the GREAT and PIMC cohorts, including smoking, alcohol use, and drug use. One explanation for this may be variance in disease severity across different samples of previous studies, as patients with more severe D-M have been found to have a higher prevalence of comorbid substance abuse or dependence, possibly indicating self-medication (Angst et al., 2019; Pfohl et al., 1982). Our results from the PIMC dataset support the above explanation to some extent as the D-M group showed more frequent psychiatric hospitalization and more medical intervention when compared with the UM group. We found that personality disorders were less frequent in the UM group than in the D-M group. We are aware of only one prior study that has reported similar proportions of comorbid axis II disorders (including OCD and personality disorders) in the UM and D-M groups (Baek et al., 2014). To summarize, the present study demonstrated overall differences in patterns of comorbidity between UM and D-M patients. D-M patients were found to have a generally increased risk of comorbid psychiatric and physical diseases to a significantly greater degree than UM patients.

To further characterize the UM group, we examined differences in the psychosocial characteristics of our UM and D-M groups (Table 3). We found that the UM group was significantly more extroverted (*p* = 0.004) and less neurotic (*p* < 0.001) than the D-M group, which echo findings in prospective studies showing high neuroticism to predict future depression, and more extraversion to predict the risk of mania (Barnett et al., 2011; Lozano & Johnson, 2001; Sparding, Pålsson, Joas, Hansen, & Landén, 2017). Moreover, previous studies observed that extraversion is positively correlated with hyperthymic temperament and neuroticism is positively correlated with depressive temperament (Blöink, Brieger, Akiskal, & Marneros, 2005; Rózsa et al., 2008). Patients with UM have been reported to exhibit hyperthymic temperaments (Angst & Grobler, 2015). Temperament is thought to be shaped by both heredity and experiences with a strong biological basis. The UM and D-M groups demonstrated distinct personality profiles, offering partial support for the notion that UM may be an entirely separate condition to D-M (Angst & Grobler, 2015). In our measures of sleep characteristics and chronotypes, the prevailing chronotype exhibited a difference between the UM and D-M groups (*p* = 0.041). More patients in the UM group belonged to the morningness type than those in the D-M group (28.57% *v.* 16.85%, *p* = 0.017). A similar trend was observed in Mittal, Mehta, Solanki, Swami, and Meena (2013) study, though the proportion did not differ significantly between their UM and D-M groups (*p* = 0.43) (Mittal et al., 2013). Some previous studies also found that depression was predicted by eveningness preference (Haraden, Mullin, & Hankin, 2017; Kivelä, Papadopoulos, & Antypa, 2018). Furthermore, the chronotype preference had been reported to be associated with lithium response (Federoff et al., 2021; McCarthy et al., 2019), and lithium responsive BD patients showed greater morningness tendencies (McCarthy et al., 2019).

There were several limitations to the present study. First, the clinical GREAT study had a retrospective design, making recall bias a potential issue. Most clinical features relied on patients' recall, e.g. the number of manic episodes, age of BD onset, physical conditions, etc., which may not be completely accurate. Furthermore, the sample size of the GREAT study was moderate and not a population-representative sample. Our analyses may not have had sufficient power to detect differences between the UM and D-M groups in some clinical features. The PIMC study used population-based medical records, avoiding such recall bias, and providing more accurate information on physical conditions. However, PIMC data may represent more severe BD patients, and detailed clinical features could not be obtained for the PIMC cohort or could only be imperfectly inferred from the dataset (e.g. numbers of episodes, age of onset). Second, hypomania was not included in both datasets. Third, the average age in our clinical sample was 40 years, whereas the average age at the end of the study in the PIMC cohort was 48 years. In both the UM and D-M groups, the younger sample will have been less likely to have developed chronic physical conditions than older patients. This may have led to underestimations in the GREAT study of associations between UM/D-M status and physical comorbidities. Nonetheless, we used the PIMC cohort for verification of our effect estimations. Forth, the NHIRD was not implemented until 1995 so medical information from earlier than 1995 was not available. This means that for some patients, their age when first diagnosed could be inaccurate. The proportion of UM found might also be higher than it might with longer follow-up periods. We endeavored to minimize such effects by selecting patients diagnosed between 2000 and 2002 and following them up until the end of 2011. This ensured that the follow-up durations in the GREAT and PIMC studies were similar. Last, there was potential for misclassification of UM using drug treatment information in the PIMC dataset. Patients who are responsive to lithium and lack of antidepressant use may still show both poles of the disorder. There was one-third (D-M group) to one-half (UM group) of patients prescribed with adequate doses of lithium. We performed a sensitivity analysis to remove patients with adequate doses of lithium, and the proportion of UM decreased to 12.68%. Therefore, the proportion of UM was quite robust, ranged from 12.68% to 14.87% in the PIMC cohort. On the other hand, patients may receive low-dose antidepressants (e.g. duloxetine and imipramine are commonly prescribed) for treating chronic pain in clinical practice in Taiwan. Therefore, our definition of UM group allowed the low-dose usage (less than 28 DDD in consecutive 28 days) of antidepressants or lamotrigine.

In conclusion, the present study provided the first line of evidence for a more comprehensive evaluation of UM and D-M in terms of clinical characteristics and lifetime comorbidity of physical illnesses and psychiatric disorders in the Taiwanese population using samples of two cohorts. Our findings provide a better understanding of the heterogeneity among subgroups of bipolar illness. The proportion of UM is estimated to be 12.9–14.9% among patients diagnosed with BD. UM patients are likely to experience more psychosis and less suicidal thoughts and behaviors. They are significantly more extroverted and less neurotic, are more inclined to the morningness chronotype, and experience better sleep quality than standard D-M patients. Our results also demonstrated differences in the lifetime comorbidity patterns of chronic physical diseases and psychiatric disorders between patients with UM and D-M, especially cardiovascular diseases and anxiety disorders. These results suggest the need for further research into the underlying mechanisms and differences between UM and D-M.

## Supplementary material

For supplementary material accompanying this paper visit https://doi.org/10.1017/S0033291722000782. click here to view supplementary material
