# Supplementary material for: Characterization of clinical features and comorbidities between bipolar affective disorder with and without depressive episodes
Source: Psychol Med. 2022 Mar 24;53(9):4103–13. doi: 10.1017/S0033291722000782 (PMC10317814; doi:10.1017/S0033291722000782)
Supplement: Supplementary file 1 [file S0033291722000782sup001.pdf]

Supplementary Materials

Table S1. Adjusted odds ratio of lifetime comorbidity conditions between UM and D-M groups

Comorbidities	GREAT study			PIMC cohort		
	PM (n=131, 12.91%)	D-M (n=884, 87.09%)	OR_{adj} (95% CI)	PM (n=1241, 14.87%)	D-M (n=7102, 85.13%)	OR_{adj} (95% CI)
**Physical diseases, n (%)**						
Metabolic disease						
Diabetes mellitus	13 (9.92)	50 (5.79)	0.69 (0.35-1.36)	240 (19.34)	1686 (23.74)	1.29 (1.10-1.51)[**]
Hyperlipidemia	-	-	-	245 (19.74)	2191 (30.85)	1.82 (1.56-2.12)[**]
Thyroid disease	8 (6.11)	47 (5.45)	0.87 (0.39-1.93)	81 (6.53)	800 (11.26)	1.66 (1.31-2.11)[**]
Cerebrovascular disease	0 (0.00)	6 (0.69)	-	127 (10.23)	1223 (17.22)	2.03 (1.64-2.52)[**]
Cardiovascular disease						
Hypertension	15 (11.45)	85 (9.85)	1.10 (0.59-2.05)	330 (26.59)	2493 (34.34)	1.58 (1.35-1.85)[**]
Myocardial infarction	0 (0.00)	21 (2.43)	-	8 (0.64)	74 (1.04)	1.69 (0.81-3.55)
Coronary artery disease	3 (2.29)	64 (7.38)	3.33 (1.03-10.78)[*]	143 (11.52)	1400 (19.71)	2.03 (1.66-2.48)[**]
Renal disease	2 (2.35)	23 (5.09)	2.53 (0.58-11.06)	89 (7.17)	893 (12.57)	1.88 (1.49-2.37)[**]
Respiratory disease						
COPD	1 (2.17)	4 (0.98)	0.46 (0.05-4.19)	164 (13.22)	1538 (21.66)	1.93 (1.61-2.32)[**]
Asthma	10 (7.63)	68 (7.83)	0.98 (0.49-1.97)	128 (10.31)	1146 (16.14)	1.62 (1.33-1.97)[**]
Gastrointestinal disease						
Peptic ulcer disease	3 (6.52)	34 (8.31)	1.24 (0.36-4.23)	214 (17.24)	2275 (32.03)	2.32 (1.98-2.72)[**]
Irritable bowel syndrome	-	-	-	94 (7.57)	1318 (18.56)	2.69 (2.16-3.35)[**]
Constipation	8 (9.41)	121 (26.77)	3.35 (1.56-7.17)[**]	358 (28.85)	3531 (49.72)	2.37 (2.06-2.71)[**]
Autoimmune disease[a]	7 (5.38)	77 (8.96)	1.94 (0.87-4.34)	46 (3.71)	592 (8.34)	2.17 (1.60-2.96)[**]
Neurological disease						
Epilepsy	2 (1.53)	22 (2.53)	1.54 (0.36-6.65)	63 (5.08)	917 (12.91)	2.97 (2.28-3.87)[**]

**Psychiatric disorders, n (%)**						
Personality disorder	-	-	-	39 (3.14)	1318 (18.56)	7.27 (5.25-10.08)**
Anxiety disorder[b]	-	-	-	257 (20.71)	3721 (52.39)	4.10 (3.54-4.75)**
GAD	3 (6.67)	53 (12.65)	1.98 (0.59-6.63)	33 (2.66)	814 (11.46)	4.52 (3.17-6.44)**
Panic disorder	1 (2.27)	64 (16.49)	8.46 (1.14-62.69)*	8 (0.64)	634 (8.93)	14.38 (7.14-28.97)**
Phobic disorder	2 (4.65)	103 (26.89)	7.54 (1.79-31.79)**	3 (0.24)	178 (2.51)	10.65 (3.40-33.40)**
OCD	-	-	-	6 (0.48)	451 (6.35)	14.18 (6.32-31.81)**
Substance use disorder[c]	6 (13.04)	105 (24.82)	2.51 (1.00-6.29)*	123 (9.91)	1714 (24.13)	3.23 (2.65-3.93)**

COPD, Chronic obstructive pulmonary disease; GAD, Generalized anxiety disorder; OCD, Obsessive-compulsive disorder.
All diseases included missing value.
The odd ratio estimation adjusted for sex and age in GREAT and adjusted for sex and age at the end of the study in the PIMC cohort.
* p-value ≤ 0.05; ** p < 0.01
[a] Autoimmune disease included arthritis and rheumatism.
[b] Anxiety disorder in PIMC cohort contained all types of anxiety disorders.
[c] Substance use disorder in GREAT contained smoking, alcohol use, and drug use.
